# Supplementary material for: Comprehensive Metabolite Profiling of Four Different Beans Fermented by Aspergillus oryzae
Source: Molecules. 2022 Nov 16;27(22):7917. doi: 10.3390/molecules27227917 (PMC9695057; doi:10.3390/molecules27227917)
Supplement: Supplementary file 1 [file molecules-27-07917-s001.zip › molecules-2019362-supplementary.pdf]

Comprehensive Metabolite Profiling of Four Different Beans Fermented by *Aspergillus Oryzae*

Yeon Hee Lee [1], Na-Rae Lee [2,*] and Choong Hwan Lee [1,2,*]

[1] Department of Bioscience and Biotechnology, Konkuk University, Seoul 05029, Korea

[2] Research Institute for Bioactive-Metabolome Network, Konkuk University, Seoul 05029, Korea

* Correspondence: michelle3690@gmail.com (N.R.L.); chlee123@konkuk.ac.kr (C.H.L.); Tel.: +82-2-2049-6177 (C.H.L.)

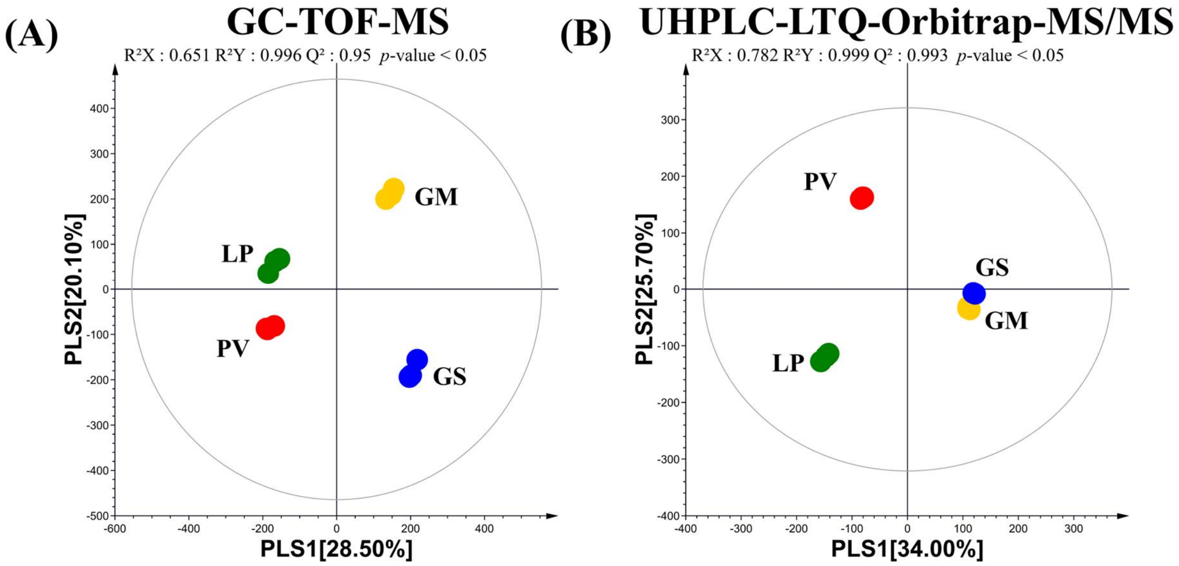

Figure S1. Partial least-square discriminant analysis of the metabolites in the four beans before fermenting. The methods included GC-TOF-MS (A) and UHPLC-LTQ-Orbitrap-MS/MS (B). (● (GM): *Glycine max*, ● (GS): *Glycine soja*, ● (PV): *Phaseolus vulgaris*, ● (LP): *Lablab purpureus*).

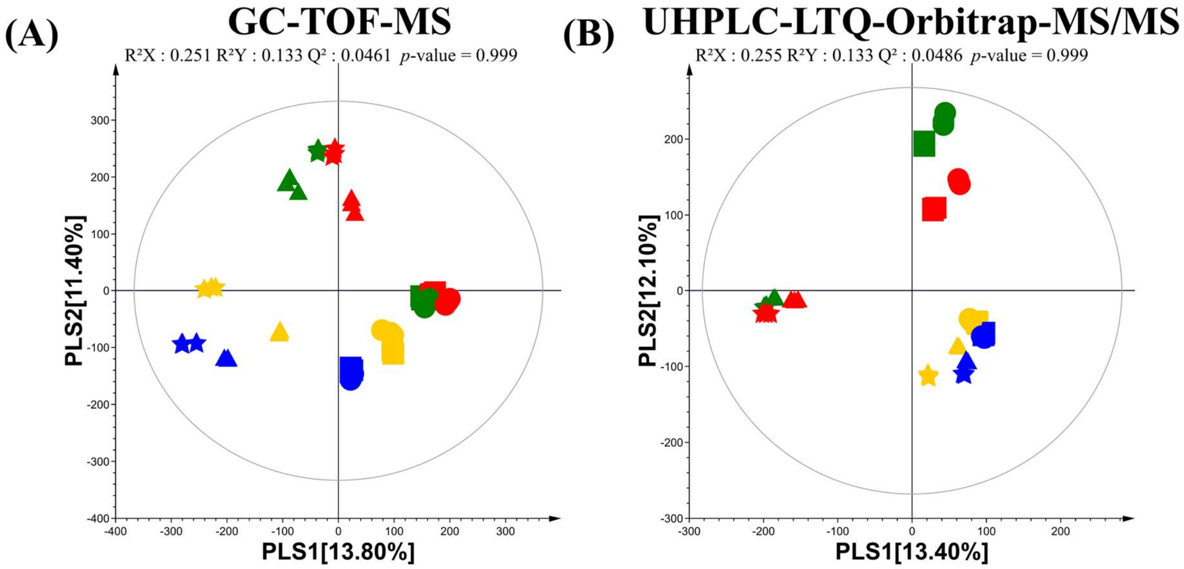

Figure S2. Partial least-square discriminant analysis of the metabolites in the four beans that were fermented with *Aspergillus oryzae*. The methods included GC-TOF-MS (A) and UHPLC-LTQ-Orbitrap-MS/MS (B). GM: *Glycine max*, GS: *Glycine soja*, PV: *Phaseolus vulgaris*, LP: *Lablab purpureus*, 0D: 0 day after fermentation, 1D: 1 day after fermentation, 2D: 2 days after fermentation, 3D: 3 days after fermentation

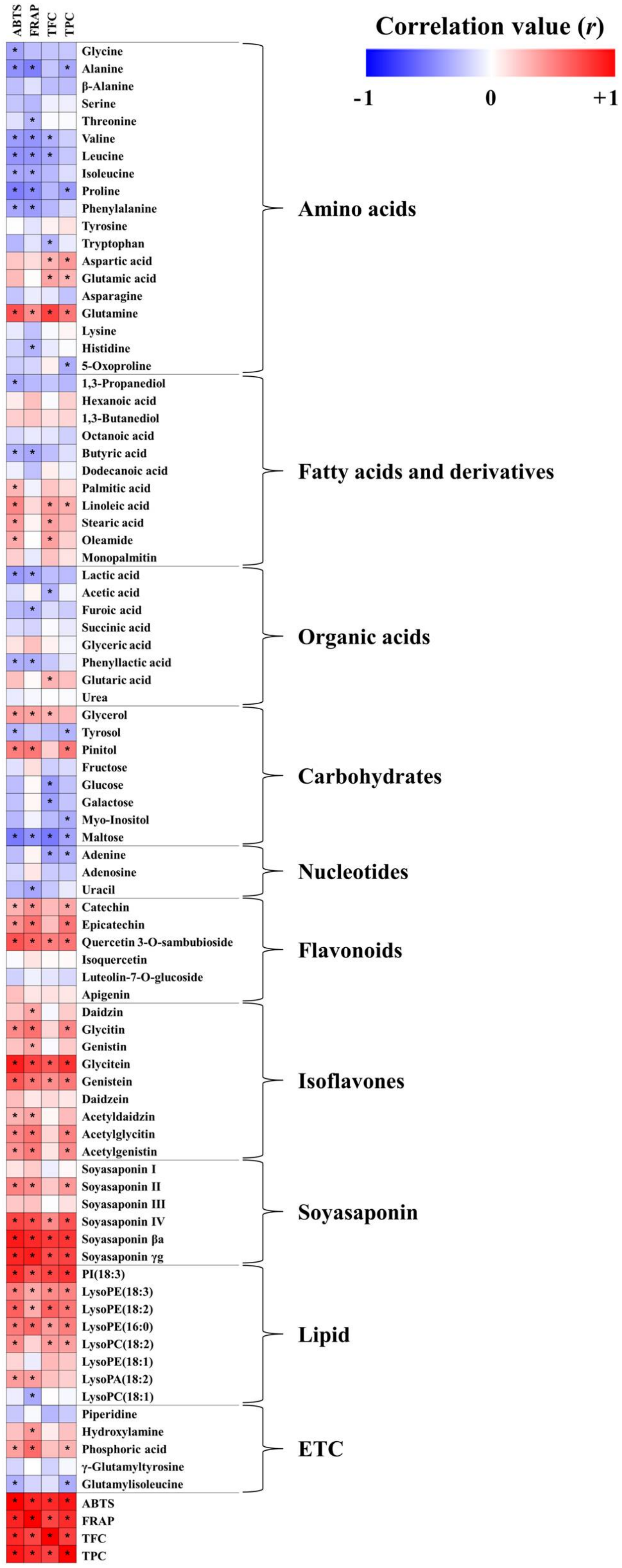

Figure S3. Correlation map between the metabolite levels and antioxidant activities. Each metabolite is identified as a significantly different metabolite through PLS-DA. Each square indicates Pearson's correlation coefficient values (r) of a pair of metabolites and assayed activities. The blue color represents a negative correlation ($-1 < r < 0$) and red color represents a positive correlation ($0 < r < 1$). Asterisks indicate significant difference ($p < 0.05$).

Table S1. Discriminative metabolites in the four beans from the PLS-DA model of the GC-TOF-MS data.

No.	Tentative identification	RT(min)	VIP 1	VIP 2	Unique Mass(m/z)	Mass Fragment pattern(m/z)	*p*-value	ID
Amino acids								
1	Alanine	5.76	1.08	0.78	116	116 73 75 117 103 74 59 190 100 118	0.00	STD/MS
2	Valine	6.98	1.76	1.24	144	144 73 218 145 147 100 75 74 59 146	0.00	STD/MS
3	Leucine	7.53	1.76	1.25	158	158 159 102 100 160 232 260 142 86 115	0.00	STD
4	Isoleucine	7.75	1.45	1.33	158	158 73 218 159 100 74 160 232 219 59 86	0.00	STD/MS
5	Proline	7.81	1.86	1.32	142	142 73 143 147 216 144 74 59 66 72	0.00	STD/MS
6	Glycine	7.89	1.14	0.91	174	174 90 86 175 146 100 248 176 130 59	0.00	STD/MS
7	Serine	8.38	0.95	1.25	204	73 204 218 147 100 205 75 219 74 188	0.00	STD/MS
8	Threonine	8.63	1.80	1.31	117	73 117 218 219 101 147 57 75 291 74	0.00	STD/MS
9	β-Alanine	8.97	0.29	1.24	174	174 248 86 290 175 249 133 59 130 250	0.00	MS
10	Aspartic acid	9.76	1.02	1.27	232	73 232 100 147 75 218 74 233 202 188	0.00	STD/MS
11	5-Oxoproline	9.83	1.84	1.33	156	156 73 147 75 84 157 230 258 74 158	0.00	MS
12	Phenylalanine	10.02	1.77	1.25	120	120 146 75 91 130 121 74 103 77 65	0.00	STD
13	Glutamic acid	10.54	1.04	1.47	246	73 246 128 147 75 156 247 84 74 230	0.00	STD
14	Asparagine	10.96	1.34	0.98	116	73 116 75 132 231 147 74 141 188 100	0.00	STD/MS
15	Glutamine	11.71	1.31	0.97	245	156 155 75 245 157 128 131 203 114 145	0.03	STD
16	Lysine	12.73	0.03	1.54	317	73 174 317 156 128 175 230 59 318 147	0.00	MS
17	Histidine	12.77	0.58	0.77	154	154 254 155 100 255 156 153 82 356 256	0.00	STD
18	Tyrosine	12.87	0.84	1.46	218	218 219 100 280 220 179 281 132 180 354	0.00	STD/MS
Fatty acids and derivatives								
19	1,3-Propanediol	5.21	0.43	1.41	115	147 73 130 115 66 148 59 177 131 149	0.00	MS
20	Hexanoic acid	5.39	1.85	1.31	173	75 73 173 117 131 132 74 76 55 61	0.00	MS
21	1,3-Butanediol	5.51	1.20	1.08	117	73 147 117 75 129 103 148 133 74	0.05	MS
22	Octanoic acid	7.40	1.27	1.41	201	201 117 55 129 69 202 70 67 99 143	0.00	MS
23	Butyric acid	8.99	1.19	1.38	233	73 233 143 75 117 147 71 234 145 144	0.00	MS
24	Dodecanoic acid	10.73	1.16	1.37	257	75 117 129 55 132 57 131 145 257 76	0.00	MS
25	Palmitic acid	13.44	1.19	1.16	173	73 117 75 132 313 129 55 145 57 69	0.00	STD/MS
26	Linoleic acid	14.47	1.49	1.19	337	73 75 55 67 81 117 95 129 69 54	0.00	STD/MS
27	Stearic acid	14.61	0.47	0.98	341	117 341 145 202 119 356 301 203 95	0.00	STD/MS
28	Oleamide	15.62	1.08	0.81	131	75 131 73 144 116 128 55 54 115 145	0.00	MS
29	Monopalmitin	16.50	0.17	1.51	371	73 147 371 57 55 129 75 71 117 103	0.00	MS
Organic acids								
30	Lactic acid	5.29	1.11	0.85	117	73 147 117 191 66 148 75 190 74 59	0.00	STD/MS
31	Acetic acid	5.45	1.84	1.31	66	73 147 66 148 75 177 205 74 133 149	0.00	MS
32	Furoic acid	5.45	0.97	1.40	125	125 95 169 126 184 170 67 96 97 85	0.00	MS
33	Succinic acid	7.90	0.37	1.11	247	147 75 247 148 73 55 56 149 129 172	0.00	STD/MS
34	Glyceric acid	8.10	1.33	0.95	292	73 147 189 103 292 133 75 117 102 74	0.00	STD/MS
35	Glutaric acid	10.42	0.05	0.42	247	73 147 75 247 115 231 199 273 74 109	0.00	MS
36	Urea	7.16	1.26	1.27	171	171 147 73 189 99 172 100 74 148 173	0.00	STD/MS
Carbohydrates and derivatives								
37	Glycerol	7.55	1.22	1.31	205	73 147 205 117 103 133 206 218 148 204	0.00	MS
38	Pinitol	12.22	0.80	1.38	217	73 147 217 260 133 191 318 159 247 129	0.00	MS
39	Fructose	12.48	1.00	0.75	307	73 103 217 147 307 74 133 75 117 218	0.00	STD/MS
40	Glucose	12.67	1.15	1.40	205	73 205 319 147 160 103 217 320 117 206	0.00	STD/MS
41	Galactose	12.67	0.38	1.17	319	73 205 319 147 160 103 217 320 117 206	0.00	STD/MS
42	Myo-Inositol	13.92	1.67	1.37	305	73 217 147 305 191 318 204 306 129 265	0.00	STD/MS
43	Maltose	17.69	0.51	1.43	361	73 361 204 147 217 103 205 362 129 117	0.00	STD
Nucleotides								
44	Adenine	12.43	1.05	1.12	264	264 279 265 96 266 87 174 113 97 125	0.00	MS
45	Adenosine	16.87	0.25	1.53	236	73 236 230 217 245 103 147 192 75 74	0.00	MS
46	Uracil	8.19	0.10	1.48	241	73 241 147 99 245 256 255 75 113 242	0.00	STD/MS
Etc.								
47	Piperidine	4.06	1.14	1.37	142	142 156 73 157 59 86 84 143 116 114	0.00	MS
48	Hydroxylamine	5.92	0.69	1.40	133	73 133 146 119 147 59 249 130 86 74	0.00	MS
49	Phosphoric acid	7.57	1.39	1.20	299	299 73 300 314 301 133 207 193 283 211	0.00	MS

Table S2. Discriminative metabolites in the four beans from the PLS-DA model of the UHPLC-LTQ-Orbitrap-MS/MS data.

No.	Tentative Identification	Rt (min)	VIP 1	VIP 2	[M–H]-	[M+H]+	M.W.	MS^n Fragments (m/z)	*p*-value	Formula	RBD	Error (ppm)	Ref
Flavonoid													
1	Catechin	3.69	0.34	1.34	289.0718	291.0866	290	289>245>203>175	0.00	C15H14O6	9.5	-0.143	[42]
2	Epicatechin	4.20	0.26	1.07	289.0721	291.0864	290	289>245>203>175	0.00	C15H14O6	9.5	1.102	[41]
3	Quercetin 3-O-sambubioside	4.54	1.54	1.22	595.1289	619.1291(Na)	596	595>371>327,265>146,138	0.00	C26H28O16	13.5	-2.601	[43]
4	Isoquercetin	4.92	1.63	1.18	463.0889	465.1028	464	463>301>178>150	0.00	C21H20O12	12.5	1.513	[44]
5	Luteolin-7-O-glucoside	5.16	0.67	1.36	447.0941	449.1080	448	447>285>241>213	0.00	C21H20O11	12.5	1.869	[41]
6	Apigenin	6.44	1.25	0.93	269.0458	271.0601	270	269>225>197>169	0.00	C15H10O5	11.5	0.979	[41]
Isoflavones													
7	Daidzin	4.54	1.61	1.18	415.1029	417.1179	416	415>253>223>195	0.00	C21H20O9	12.5	-1.314	[29]
8	Glycitin	4.66	1.66	1.20	445.1143	447.1284	446	445>283>268>240	0.00	C22H22O10	12.5	0.607	[29]
9	Genistin	5.03	1.63	1.19	431.0978	433.1130	432	431>268>239>211	0.00	C21H20O10	12.5	-1.229	[25]
10	Glycitein	5.08	1.39	0.99	283.0613	285.0757	284	283>268>240>196	0.00	C16H12O5	11.5	0.188	[25]
11	Genistein	5.43	1.65	1.20	269.0458	271.0600	270	269>225>181	0.00	C15H10O5	11.5	0.756	[25]
12	Daidzein	5.79	1.14	0.86	253.0508	255.0653	254	253>209>141	0.00	C15H10O4	11.5	0.822	[25]
13	Acetyldaidzin	5.34	1.66	1.21	457.1135	459.1285	458	457>252>223>194	0.00	C23H22O10	13.5	-1.072	[5]
14	Acetylglycitin	5.40	1.63	1.18	487.1246	489.1393	488	487>468>267>223	0.00	C24H24O11	13.5	0.093	[5]
15	Acetylgenistin	5.80	1.63	1.18	473.1095	475.1231	474	473>268>224>180	0.00	C23H22O11	13.5	1.195	[29]
Soyasaponin													
16	Soyasaponin I	7.21	0.67	1.18	941.5089	943.5255	942	941>923>879>733	0.00	C48H78O18	10.5	-2.856	[25]
17	Soyasaponin II	7.34	1.66	1.21	911.4991	913.5159	912	911>615,893>849>703	0.00	C47H76O17	10.5	-2.078	[25]
18	Soyasaponin III	7.41	1.27	1.09	795.4525	797.4667	796	795>615>457>437	0.00	C42H68O14	9.5	-1.483	[29]
19	Soyasaponin IV	7.49	1.62	1.17	765.4426	767.4576	766	765>615>457,533>437,507	0.00	C41H66O13	9.5	-0.568	[25]
20	Soyasaponin βa	7.80	1.47	1.05	1037.5324	1039.5496	1038	1037>937>641>525	0.00	C53H82O20	13.5	-0.306	[25]
21	Soyasaponin γg	7.88	1.70	1.21	921.4849	923.5006	922	921>821>641>464	0.00	C48H74O17	12.5	-0.503	[20]
Lipid													
22	PI(18:3)	7.66	0.73	1.11	593.2722	595.2875	594	593>315>152,222>78	0.00	C27H47O12P	5.5	-1.713	[45]
23	LysoPE(18:3)	8.07	0.02	1.33	474.2613	476.2766	475	474>277>233>191	0.00	C23H42NO7P	4.5	-2.725	[46]
24	LysoPE(18:2)	8.48	1.19	1.06	476.2778	478.2921	477	476>279>261>243	0.00	C23H44NO7P	3.5	-0.971	[25]
25	LysoPE(16:0)	8.80	1.07	0.77	452.2774	454.2918	453	452>255>237>83	0.00	C21H44NO7P	1.5	-1.973	[25]
26	LysoPC(18:2)	8.83	1.10	1.16	504.3091	520.3389	505	504>279>261>243	0.00	C26H50NO7P	2.5	-1.722	[25]
27	LysoPE(18:1)	9.00	0.62	1.34	478.2941	480.3080	479	478>281>263>245	0.00	C23H46NO7P	2.5	0.392	[25]
28	LysoPA(18:2)	9.00	1.06	0.86	433.2354	435.2502	434	433>153>78	0.00	C21H39O7P	3.5	-1.646	[41]
29	LysoPC(18:1)	9.49	0.84	0.91	506.3245	522.3544	507	506>281>263>95	0.00	C26H52NO7P	1.5	-1.887	[25]
30	Linoleamide	10.20	-	-	-	280.2622	279	(+)280>263>245>161	0.00	C18H33NO	2.5	-2.823	[39]
31	Oleamide	10.78	-	-	-	282.2773	281	(+)282>265>247>149	0.00	C18H35NO	1.5	-4.22	[29]
Etc													
32	γ-Glutamyltyrosine	1.39	1.69	1.22	309.1088	311.1235	310	309>291,127>83	0.00	C14H18N2O6	7.5	-1.228	[40]
33	Glutamylisoleucine	2.43	0.24	0.84	259.1307	261.1435	260	259>128>84	0.00	C11H20N2O5	3.5	-0.482	[40]

Table S3. Discriminative metabolites in the four beans that were fermented with *Aspergillus oryzae* from the PLS-DA model of the GC-TOF-MS data.

No.	Tentative identification	RT(min)	VIP 1	VIP 2	Unique Mass(m/z)	Mass Fragment pattern(m/z)	*p*-value	ID
Amino acids								
1	Alanine	5.76	0.19	1.17	116	116 73 75 117 103 74 59 190 100 118	0.00	STD/MS
2	Valine	6.98	0.20	2.00	144	144 73 218 145 147 100 75 74 59 146	0.00	STD/MS
3	Leucine	7.53	0.08	1.95	158	158 159 102 100 160 232 260 142 86 115	0.00	STD
4	Isoleucine	7.75	0.19	1.86	158	158 73 218 159 100 74 160 232 219 59 86	0.00	STD/MS
5	Proline	7.81	0.95	1.60	142	142 73 143 147 216 144 74 59 66 72	0.00	STD/MS
6	Glycine	7.89	0.48	1.31	174	174 90 86 175 146 100 248 176 130 59	0.00	STD/MS
7	Serine	8.38	0.61	1.42	204	73 204 218 147 100 205 75 219 74 188	0.00	STD/MS
8	Threonine	8.63	1.00	1.60	117	73 117 218 219 101 147 57 75 291 74	0.00	STD/MS
9	β-Alanine	8.97	0.73	0.63	174	174 248 86 290 175 249 133 59 130 250	0.00	MS
10	Aspartic acid	9.76	1.29	1.12	232	73 232 100 147 75 218 74 233 202 188	0.00	STD/MS
11	5-Oxoproline	9.83	0.93	0.66	156	156 73 147 75 84 157 230 258 74 158	0.00	MS
12	Phenylalanine	10.02	0.47	1.93	120	120 146 75 91 130 121 74 103 77 65	0.00	STD
13	Glutamic acid	10.54	2.25	1.85	246	73 246 128 147 75 156 247 84 74 230	0.00	STD
14	Asparagine	10.96	1.50	1.06	116	73 116 75 132 231 147 74 141 188 100	0.00	STD/MS
15	Glutamine	11.71	2.41	1.72	245	156 155 75 245 157 128 131 203 114 145	0.00	STD
16	Lysine	12.73	1.30	1.86	317	73 174 317 156 128 175 230 59 318 147	0.00	MS
17	Histidine	12.77	0.97	1.73	154	154 254 155 100 255 156 153 82 356 256	0.00	STD
18	Tyrosine	12.87	1.24	1.58	218	218 219 100 280 220 179 281 132 180 354	0.00	STD/MS
19	Tryptophan	14.67	0.95	1.04	202	202 73 203 204 291 74 75 218 147 100	0.00	STD/MS
Fatty acids and derivatives								
20	1,3-Propanediol	5.21	1.31	0.95	115	147 73 130 115 66 148 59 177 131 149	0.00	MS
21	Octanoic acid	7.40	0.93	0.67	201	201 117 55 129 69 202 70 67 99 143	0.00	MS
22	Butyric acid	8.99	0.45	1.85	233	73 233 143 75 117 147 71 234 145 144	0.00	MS
23	Dodecanoic acid	10.73	0.90	1.23	257	75 117 129 55 132 57 131 145 257 76	0.00	MS
24	Palmitic acid	13.44	2.11	1.50	173	73 117 75 132 313 129 55 145 57 69	0.00	STD/MS
25	Linoleic acid	14.47	2.23	1.64	337	73 75 55 67 81 117 95 129 69 54	0.00	STD/MS
26	Stearic acid	14.61	2.25	1.60	341	117 341 145 202 119 356 301 203 95	0.00	STD/MS
27	Oleamide	15.62	1.54	1.16	131	75 131 73 144 116 128 55 54 115 145	0.00	MS
28	Monopalmitin	16.50	1.70	1.25	371	73 147 371 57 55 129 75 71 117 103	0.00	MS
Organic acids								
29	Lactic acid	5.29	0.81	1.51	117	73 147 117 191 66 148 75 190 74 59	0.00	STD/MS
30	Acetic acid	5.45	1.33	1.23	66	73 147 66 148 75 177 205 74 133 149	0.00	MS
31	Furoic acid	5.45	0.39	1.09	125	125 95 169 126 184 170 67 96 97 85	0.00	MS
32	Succinic acid	7.90	0.32	1.57	247	147 75 247 148 73 55 56 149 129 172	0.00	STD/MS
33	Glyceric acid	8.10	0.22	1.14	292	73 147 189 103 292 133 75 117 102 74	0.00	STD/MS
34	Phenyllactic acid	10.32	0.24	1.59	193	193 57 71 85 194 220 55 163 267 70	0.00	MS
35	Glutaric acid	10.42	1.94	1.62	247	73 147 75 247 115 231 199 273 74 109	0.00	MS
36	Urea	7.16	0.82	0.68	171	171 147 73 189 99 172 100 74 148 173	0.00	STD/MS
Carbohydrates and derivatives								
37	Glycerol	7.55	0.00	1.30	205	73 147 205 117 103 133 206 218 148 204	0.00	MS
38	Tyrosol	10.17	1.23	0.94	179	179 180 103 282 193 283 267 213 213 70 253	0.00	MS
39	Pinitol	12.22	0.54	1.57	217	73 147 217 260 133 191 318 159 247 129	0.00	MS
40	Fructose	12.48	1.61	1.60	307	73 103 217 147 307 74 133 75 117 218	0.00	STD/MS
41	Glucose	12.67	1.88	1.48	205	73 205 319 147 160 103 217 320 117 206	0.00	STD/MS
42	Galactose	12.67	1.83	1.46	319	73 205 319 147 160 103 217 320 117 206	0.00	STD/MS
43	Myo-Inositol	13.92	2.08	1.76	305	73 217 147 305 191 318 204 306 129 265	0.00	STD/MS
44	Maltose	17.69	0.52	1.74	361	73 361 204 147 217 103 205 362 129 117	0.00	STD
Nucleotides								
45	Adenine	12.43	1.53	1.22	264	264 279 265 96 266 87 174 113 97 125	0.00	MS
46	Adenosine	16.87	1.62	1.35	236	73 236 230 217 245 103 147 192 75 74	0.00	MS
47	Uracil	8.19	0.64	1.96	241	73 241 147 99 245 256 255 75 113 242	0.00	STD/MS
Etc.								
48	Piperidine	4.06	1.42	1.31	142	142 156 73 157 59 86 84 143 116 114	0.00	MS
49	Hydroxylamine	5.92	0.44	1.23	133	73 133 146 119 147 59 249 130 86 74	0.00	MS
50	Phosphoric acid	7.57	0.80	1.43	299	299 73 300 314 301 133 207 193 283 211	0.00	MS

Table S4. Discriminative metabolites in the four beans that were fermented with *Aspergillus oryzae* from the PLS-DA model of the UHPLC-LTQ-Orbitrap-MS/MS data.

No.	Tentative Identification	Rt (min)	VIP 1	VIP 2	[M–H]-	[M+H]+	M.W.	MS^n Fragments (m/z)	*p*-value	Formula	RBD	Error (ppm)	Ref
Flavonoid													
1	Catechin	3.69	0.90	0.77	289.0718	291.0866	290	289>245>203>175	0.00	C15H14O6	9.5	-0.143	[42]
2	Epicatechin	4.20	1.17	0.83	289.0721	291.0864	290	289>245>203>175	0.00	C15H14O6	9.5	1.102	[41]
3	Quercetin 3-O-sambubioside	4.54	2.03	1.90	595.1289	619.1291(Na)	596	595>371>327,265>146,138	0.00	C26H28O16	13.5	-2.601	[43]
4	Isoquercetin	4.92	0.37	0.79	463.0889	465.1028	464	463>301>178>150	0.00	C21H20O12	12.5	1.513	[44]
5	Luteolin-7-O-glucoside	5.16	0.15	0.91	447.0941	449.1080	448	447>285>241>213	0.00	C21H20O11	12.5	1.869	[41]
6	Apigenin	6.44	1.35	1.50	269.0458	271.0601	270	269>225>197>169	0.00	C15H10O5	11.5	0.979	[41]
Isoflavones													
7	Daidzin	4.54	1.54	1.37	415.1029	417.1179	416	415>253>223>195	0.00	C21H20O9	12.5	-1.314	[29]
8	Glycitin	4.66	1.71	1.55	445.1143	447.1284	446	445>283>268>240	0.00	C22H22O10	12.5	0.607	[29]
9	Genistin	5.03	1.60	1.45	431.0978	433.1130	432	431>268>239>211	0.00	C21H20O10	12.5	-1.229	[25]
10	Glycitein	5.08	1.55	1.76	283.0613	285.0757	284	283>268>240>196	0.00	C16H12O5	11.5	0.188	[25]
11	Genistein	5.43	1.79	1.92	269.0458	271.0600	270	269>225>181	0.00	C15H10O5	11.5	0.756	[25]
12	Daidzein	5.79	1.31	1.47	253.0508	255.0653	254	253>209>141	0.00	C15H10O4	11.5	0.822	[25]
13	Acetyldaidzin	5.34	1.67	1.56	457.1135	459.1285	458	457>252>223>194	0.00	C23H22O10	13.5	-1.072	[5]
14	Acetylglycitin	5.40	1.71	1.60	487.1246	489.1393	488	487>468>267>223	0.00	C24H24O11	13.5	0.093	[5]
15	Acetylgenistin	5.80	1.66	1.53	473.1095	475.1231	474	473>268>224>180	0.00	C23H22O11	13.5	1.195	[29]
Soyasaponin													
16	Soyasaponin I	7.21	1.71	1.23	941.5089	943.5255	942	941>923>879>733	0.00	C48H78O18	10.5	-2.856	[25]
17	Soyasaponin II	7.34	1.82	1.84	911.4991	913.5159	912	911>615,893>849>703	0.00	C47H76O17	10.5	-2.078	[25]
18	Soyasaponin III	7.41	1.80	1.49	795.4525	797.4667	796	795>615>457>437	0.00	C42H68O14	9.5	-1.483	[29]
19	Soyasaponin IV	7.49	1.84	1.88	765.4426	767.4576	766	765>615>457,533>437,507	0.00	C41H66O13	9.5	-0.568	[25]
20	Soyasaponin βa	7.80	1.15	1.33	1037.5324	1039.5496	1038	1037>937>641>525	0.00	C53H82O20	13.5	-0.306	[25]
21	Soyasaponin γg	7.88	1.35	1.39	921.4849	923.5006	922	921>821>641>464	0.00	C48H74O17	12.5	-0.503	[20]
Lipid													
22	PI(18:3)	7.66	0.09	1.25	593.2722	595.2875	594	593>315>152,222>78	0.00	C27H47O12P	5.5	-1.713	[45]
23	LysoPE(18:3)	8.07	0.34	0.80	474.2613	476.2766	475	474>277>233>191	0.00	C23H42NO7P	4.5	-2.725	[46]
24	LysoPE(18:2)	8.48	0.07	1.32	476.2778	478.2921	477	476>279>261>243	0.00	C23H44NO7P	3.5	-0.971	[25]
25	LysoPE(16:0)	8.80	1.88	1.35	452.2774	454.2918	453	452>255>237>83	0.00	C21H44NO7P	1.5	-1.973	[25]
26	LysoPC(18:2)	8.83	0.47	1.24	504.3091	520.3389	505	504>279>261>243	0.00	C26H50NO7P	2.5	-1.722	[25]
27	LysoPE(18:1)	9.00	1.77	1.52	478.2941	480.3080	479	478>281>263>245	0.00	C23H46NO7P	2.5	0.392	[25]
28	LysoPA(18:2)	9.00	1.53	1.53	433.2354	435.2502	434	433>153>78	0.00	C21H39O7P	3.5	-1.646	[41]
29	LysoPC(18:1)	9.49	1.64	1.30	506.3245	522.3544	507	506>281>263>95	0.00	C26H52NO7P	1.5	-1.887	[25]
30	Linoleamide	10.20	-	-	-	280.2622	279	(+)280>263>245>161	0.00	C18H33NO	2.5	-2.823	[39]
31	Oleamide	10.78	-	-	-	282.2773	281	(+)282>265>247>149	0.00	C18H35NO	1.5	-4.22	[29]
Etc													
32	γ-Glutamyltyrosine	1.39	0.77	1.07	309.1088	311.1235	310	309>291,127>83	0.00	C14H18N2O6	7.5	-1.228	[40]
33	Glutamylisoleucine	2.43	0.43	1.54	259.1307	261.1435	260	259>128>84	0.00	C11H20N2O5	3.5	-0.482	[40]

Figure S4. Discriminative non-identification metabolites in the four beans that were fermented with *Aspergillus oryzae* from the PLS-DA model of the UHPLC-LTQ-Orbitrap-MS/MS data.

No.	Tentative Identification	Rt(min)	VIP 1	VIP 2	[M–H]-	[M+H]+	M.W.	MS^n Fragments (m/z)	*p*-value	GM				GS				PV				LP			
										0D	1D	2D	3D	0D	1D	2D	3D	0D	1D	2D	3D	0D	1D	2D	3D
1	N.I 1	1.59	1.18	1.12	255.0511	257.0677	256	255>165>135>107	0.00	0.14	0.22	0.27	0.12	5.58	5.32	3.17	1.17	0.00	0.01	0.00	0.00	0.00	0.00	0.00	0.00
2	N.I 2	2.79	2.65	1.88	295.1773	297.1922	296	295>207>179>136	0.00	0.01	0.01	0.01	0.19	0.01	0.01	0.01	0.01	0.01	0.28	3.72	4.36	0.01	0.08	2.95	4.38
3	N.I 3	3.36	1.79	1.92	303.0620	305.0770	304	303>259>187>144	0.00	1.30	1.73	1.83	1.89	2.26	2.20	2.35	2.30	0.06	0.01	0.03	0.01	0.01	0.01	0.01	0.00
4	N.I 4	3.92	1.74	1.90	305.0702	307.0930	306	305>225>181>163	0.00	1.26	1.35	1.85	2.01	2.21	2.06	2.47	2.62	0.02	0.01	0.00	0.00	0.05	0.07	0.03	0.00
5	N.I 5	4.46	0.51	1.07	225.1133	227.1279	226	225>181>163>121	0.00	0.55	0.61	1.09	0.80	0.78	0.92	2.89	3.11	0.49	0.43	0.55	0.78	0.55	0.59	0.96	0.91
6	N.I 6	4.55	1.63	1.23	268.0825	270.0972	269	268>136>92	0.00	0.12	0.12	0.12	0.12	0.12	0.52	0.12	0.12	1.42	0.12	1.93	0.12	1.43	1.49	3.15	4.98
7	N.I 7	4.73	1.56	1.10	796.4222	798.4367	797	796>778>366>322	0.00	0.00	0.00	0.00	0.02	0.00	0.00	0.00	0.00	0.00	1.80	8.63	5.50	0.00	0.01	0.01	0.01
8	N.I 8	4.80	2.42	1.72	440.2189	442.2340	441	440>180,266>146>128	0.00	0.03	0.03	0.03	0.01	0.00	0.00	0.00	0.00	0.00	0.32	5.87	4.22	0.03	0.06	2.78	2.61
9	N.I 9	4.84	1.09	1.24	493.0997	495.1133	494	493>331>316>287	0.00	2.98	2.67	2.30	1.74	0.01	0.01	2.24	3.95	0.01	0.01	0.01	0.01	0.01	0.01	0.01	0.01
10	N.I 10	5.16	1.17	1.04	515.1204	517.1343	516	515>253>225>197	0.00	0.16	0.16	0.16	0.16	3.82	2.93	4.34	1.88	0.16	0.16	0.26	0.16	1.20	0.16	0.16	0.16
11	N.I 11	5.29	1.75	1.24	627.3716	629.3873	628	(+)629>498>470>453	0.00	0.00	0.00	0.00	0.08	0.00	0.00	0.00	0.00	0.00	0.00	0.09	0.07	0.00	0.07	9.80	5.88
12	N.I 12	5.29	1.27	1.03	787.2656	789.2826	788	787>643>419>293	0.00	0.20	0.15	0.14	0.11	3.02	2.69	2.65	2.36	1.95	1.54	0.67	0.52	0.00	0.00	0.00	0.00
13	N.I 13	6.10	2.03	1.44	639.4454	641.4604	640	639>299>281>193	0.00	0.00	0.00	0.00	0.06	0.00	0.00	0.00	0.00	0.00	0.00	0.30	1.37	0.00	0.04	6.78	7.44
14	N.I 14	6.13	2.48	1.77	1013.5070	1015.5224	1014	1013>983>965>473	0.00	0.02	0.02	0.03	0.06	0.05	0.02	0.02	0.03	0.00	0.00	3.07	6.13	0.00	0.02	2.21	4.32
15	N.I 15	6.15	0.70	0.76	1089.5475	1091.5656	1090	1089>1028>878>456	0.00	0.13	6.33	3.90	3.38	0.83	0.13	0.13	0.13	0.13	0.13	0.13	0.13	0.13	0.13	0.13	0.13
16	N.I 16	6.34	0.17	1.48	823.4128	847.4096(Na)	824	823>647>485>439	0.00	0.04	0.03	0.03	0.02	0.64	0.54	0.51	0.57	0.32	0.25	0.14	0.12	5.52	3.91	1.90	1.47
17	N.I 17	6.51	1.80	1.28	701.4612	703.4754	702	701>389>371>207	0.00	0.01	0.00	0.00	0.00	0.00	0.00	0.00	0.01	0.00	0.00	0.00	0.00	0.04	0.03	7.42	8.48
18	N.I 18	6.56	0.13	1.25	955.4912	957.5050	956	955>793>613>455	0.00	0.99	1.15	1.11	0.85	0.36	0.32	0.30	0.33	0.02	0.02	0.01	0.01	4.52	3.27	1.47	1.27
19	N.I 19	6.61	1.55	1.47	523.2568	525.2707	524	523>417>311>172	0.00	0.00	0.08	2.51	1.81	0.00	0.44	1.23	1.66	0.00	0.22	2.05	1.26	0.00	0.52	2.48	1.75
20	N.I 20	6.67	0.13	1.47	809.4332	811.4482	810	809>629>567>471	0.00	0.27	0.33	0.29	0.23	0.29	0.25	0.21	0.23	0.07	0.05	0.03	0.03	5.98	4.48	1.86	1.41
21	N.I 21	6.77	0.08	1.07	1041.5380	1043.5531	1042	1041>1011>993>654,822,896	0.00	0.26	0.26	0.26	0.26	0.26	0.26	0.26	0.26	0.26	0.26	0.26	0.26	10.54	0.26	0.26	1.82
22	N.I 22	6.99	2.65	1.89	1055.5519	1057.5682	1056	1055>1025>1007>668	0.00	0.08	0.07	0.07	0.09	0.03	0.03	0.02	0.03	0.00	0.10	3.72	3.51	0.05	0.11	4.34	3.72
23	N.I 23	7.09	0.73	0.56	957.5074	959.5222	958	957>525>439>406	0.00	1.02	1.02	0.99	0.85	1.00	0.89	0.88	0.92	3.18	2.71	1.38	1.12	0.02	0.01	0.01	0.01
24	N.I 24	8.27	1.97	1.86	571.2869	573.3028	572	571>255>237>81	0.00	1.19	1.37	1.40	1.31	1.88	1.71	1.87	1.75	0.48	0.44	0.28	0.30	0.56	0.65	0.50	0.31
25	N.I 25	8.78	0.57	1.25	564.3307	566.3402	565	564>504>279>261	0.00	0.82	1.01	1.33	1.56	1.12	1.15	1.22	1.33	0.42	0.80	0.62	0.54	0.77	0.95	1.41	0.95
26	N.I 26	9.86	2.38	1.70	1038.6676	1040.6849	1039	1038>1020>1002>818	0.00	0.00	0.00	0.01	0.41	0.00	0.00	0.00	0.01	0.00	0.07	2.30	4.75	0.00	0.28	1.73	6.43
27	N.I 27	9.92	2.47	1.77	350.2699	352.2841	351	350>306>235	0.00	0.09	0.03	0.06	0.12	0.10	0.10	0.12	0.10	0.01	0.07	2.53	4.88	0.02	0.04	2.14	5.59
28	N.I 28	10.09	2.51	1.78	1052.6835	1054.7003	1053	1052>1034>1016>818	0.00	0.00	0.00	0.01	0.55	0.00	0.00	0.00	0.01	0.00	0.18	2.46	3.68	0.00	0.86	2.42	5.80
29	N.I 29	10.46	2.31	1.71	1006.6434	1008.6568	1007	1006>988>664>452	0.00	0.03	0.00	0.01	0.26	0.01	0.00	0.00	0.01	0.02	1.64	2.83	2.89	0.01	3.11	2.36	2.81
30	N.I 30	10.79	2.01	1.59	1020.6588	1022.6730	1021	1020>1002>678,984>452,762	0.00	0.03	0.00	0.01	0.12	0.01	0.00	0.00	0.01	0.02	2.06	2.83	2.34	0.01	3.79	2.30	2.46

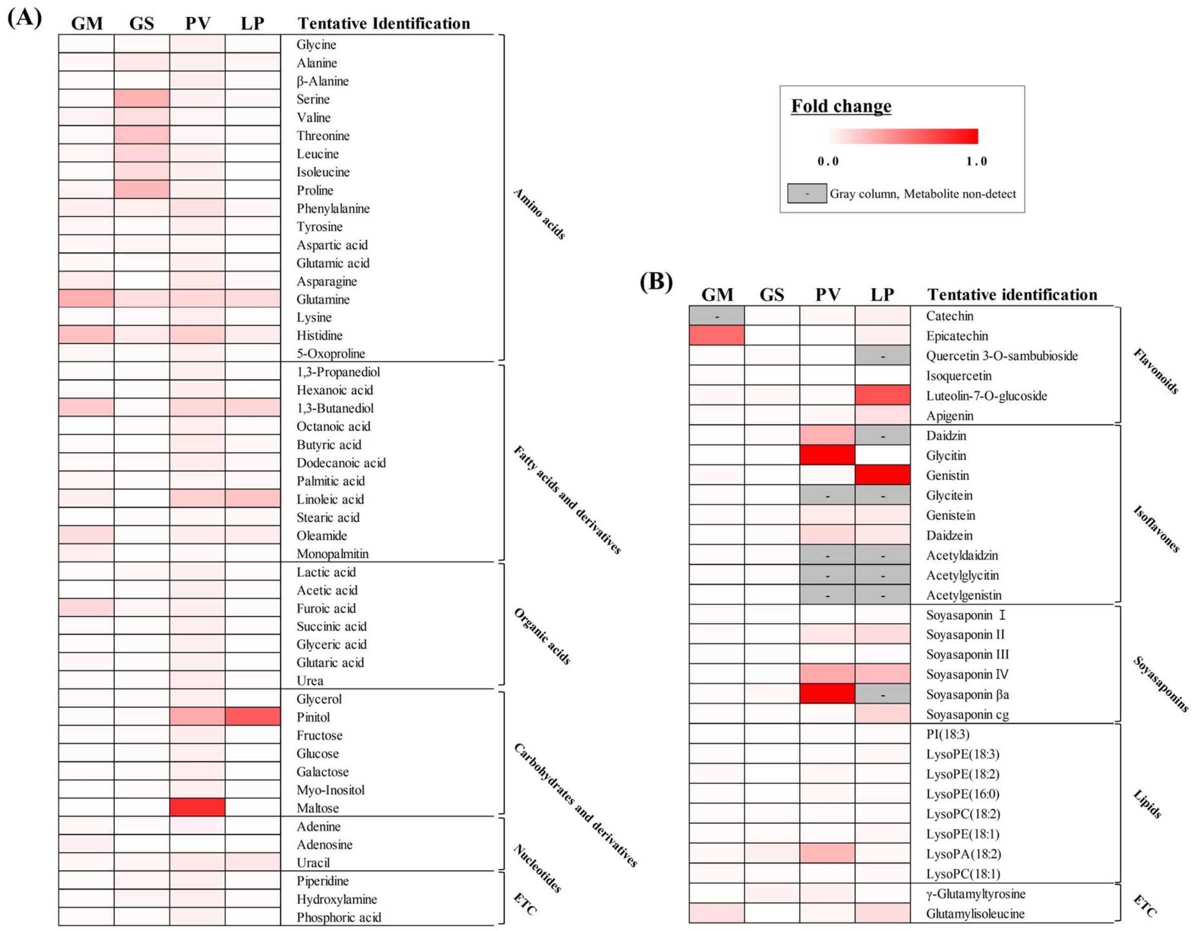

Figure S5. Heat map of the standard deviations of the relative contents of each metabolite in four different beans prior to fermentation analyzed through GC-TOF-MS (A) and UHPLC-LTQ-Orbitrap-MS/MS (B) analyses depicted in Figure 2A, B. Each square represents the magnitude of the standard deviations as a color ranging from white(0) to red(1). GM: *G. max*, GS: *G. soja*, PV: *P. vulgaris*, LP: *L. purpureus*

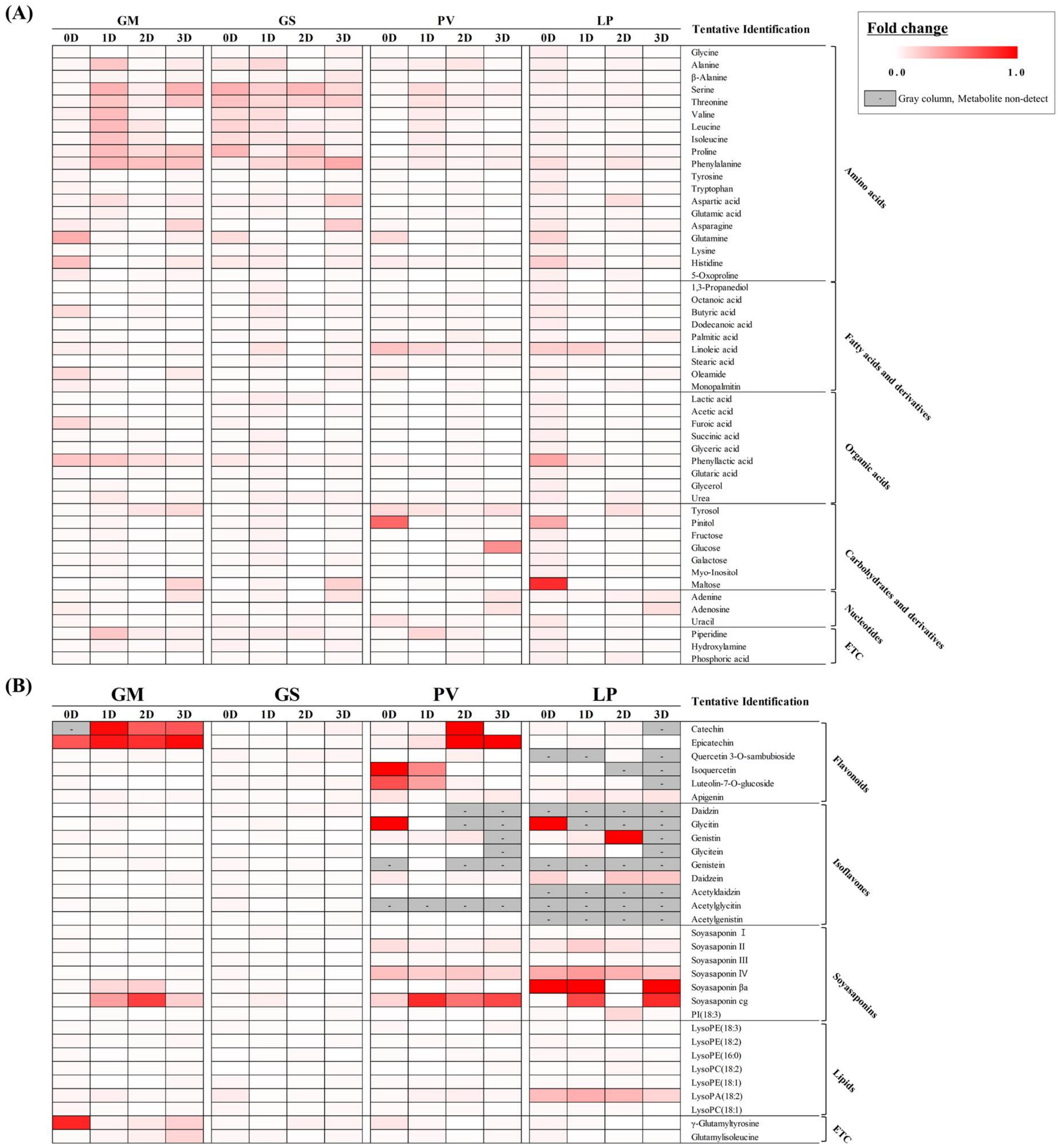

Figure S6. Heat map of the standard deviations of the relative contents of each metabolite in four different bean fermentations analyzed through GC-TOF-MS (A) and UHPLC-LTQ-Orbitrap-MS/MS (B) analyses depicted in Figure 2A, B. Each square represents the magnitude of the standard deviations as a color ranging from white(0) to red(1). GM: *G. max*, GS: *G. soja*, PV: *P. vulgaris*, LP: *L. purpureus*
